# A Glance at Some Relations of Dentistry to General Medicine

**Published:** 1897-08

**Authors:** George F. Eames

**Affiliations:** Boston, Mass.


### A Glance at Some Relations of Dentistry to General Medicine.

BY DR. GEORGE F. EAMES, BOSTON, MASS.

Abstract of paper read before the Dental Section, American Medical Association, June, 1897.

The average dentist not only fails to recognize many indications which call for systemic treatment, but fails to see his need of such recognition.

If a new drug is advertised he tries it a few times and immediately writes an article on it. He treats all forms of pyorrhea by scraping the teeth, and if the deposit continues to accumulate, the scraping is repeated. He treats sensitive dentine by the application of obtundents, and never dreams of prophylaxis, or the correction of underlying causes. During a persistent and alarming hemorrhage from the socket of a tooth, the heart is allowed to go on thumping against the chest wall, thereby resisting the local styptic and tending to increase the hemorrhage. It is still true in too many cases that a tooth is extracted because a patient asks that it be done. The dental college with its required three-year course and Latin entrance examination still continues to let loose thousands of graduates whose spelling and use of ordinary English is excruciating, and whose understanding of the principles of pathology and medicine is practically *nil.* These go out to practice upon humanity.

It has been said that the dentist's work is largely mechanical; many make it only mechanical and see only the mechanical side of it. The operator may be engaged in plugging a large distal cavity with gold, but this mechanical operation can not be disconnected from the fact that in some cases of nervous sussceptibility, the entire organism may sympathize with the local irritation to the extent of shock, or nervous collapse. What is the position of the mechanical operator in such a case? In his failure to recognize the vital connection of the teeth with the human organism he has failed to discharge his whole duty to his patient, and is guilty of criminal neglect.

A dentist may be called upon at any time to exercise his medical knowledge immediately in cases of nervous spasm, hysteria, syncope, shock, collapse, foreign bodies swallowed, poisoning, hemorrhage, etc., or in a more leisurely way in the considerations of questions relating to stomatitis, adenoid vegetations, malignant growths, operations during pregnancy, and the various inflammatory conditions of the dental pulp and pericementum.

Teeth are being lost at an alarming rate on account of the ravages of the so-called pyorrhea alveolaris, and this fact alone calls for deep, medical research, for a thorough, physical examination, including the blood, the salivary and renal secretions, followed by appropriate medical treatment. In order to do this a most thorough study of the theory and practice of medicine is absolutely required. I can see no other way to meet these pathological conditions in the mouth, believing as I do that they are the expression of some diathesis or other general disturbance. As medical specialists, we are facing the serious problem of saving thousands of teeth which at the present time are being lost in spite of the great advance which our profession has made, and it rests with us, as members of the representative medical body of the United States, to use every means in our power to secure the requirement of a broader, general and medical education as one of the essential conditions of graduation.

